# The Prognostic Value of the 31-Gene Expression Profile Test in Cutaneous Melanoma: A Systematic Review and Meta-Analysis

**DOI:** 10.3390/cancers16213714

**Published:** 2024-11-04

**Authors:** Ryan A. Durgham, Sami I. Nassar, Ramazan Gun, Shaun A. Nguyen, Ameya A. Asarkar, Cherie-Ann O. Nathan

**Affiliations:** 1 Department of Otolaryngology—Head and Neck Surgery, Medical University of South Carolina, Charleston, SC 29425, USA; durgham@musc.edu (R.A.D.); nassar@musc.edu (S.I.N.); nguyensh@musc.edu (S.A.N.); 2 Department of Otolaryngology—Head and Neck Surgery, Louisiana State University Health Sciences Center, Shreveport, LA 71103, USA; ramazan.gun@lsuhs.edu (R.G.); ameya.asarkar@lsuhs.edu (A.A.A.)

**Keywords:** cutaneous melanoma, gene expression profiling, prognostic biomarker, risk stratification, personalized medicine, meta-analysis

## Abstract

Background: Cutaneous melanoma is an increasingly common and potentially lethal form of skin cancer. Current staging systems based on clinical and pathological features have limitations in accurately predicting outcomes, particularly for early-stage disease. The 31-gene expression profile (31-GEP) test has emerged as a promising tool for improving risk stratification in melanoma patients. Methods: We conducted a systematic review and meta-analysis of studies evaluating the prognostic performance of the 31-GEP test in cutaneous melanoma. A comprehensive literature search was performed in multiple databases. Studies reporting survival outcomes stratified by 31-GEP class were included. Random-effects models were used to determine survival estimates across studies. Results: Thirteen studies comprising 14,760 patients were included in the meta-analysis. The 31-GEP test consistently stratified patients into risk groups with significantly different outcomes. The 5-year melanoma-specific survival rates were 99.8% (95% CI: 98–100%) for Class 1A, 97.6% (95% CI: 92.4–99.3%) for Class 1B/2A, and 83.4% (95% CI: 66.5–92.7%) for Class 2B. Similar trends were observed for recurrence-free and distant metastasis-free survival. Conclusions: This meta-analysis supports the prognostic utility of the 31-GEP test in cutaneous melanoma prognostication. The test consistently stratified patients into clinically meaningful risk groups across multiple survival metrics. These findings support the potential clinical utility of the 31-GEP test in enhancing current staging systems and informing personalized management strategies for melanoma patients.

## Simple Summary

Cutaneous melanoma is a malignancy of melanocytes with increasing global incidence. While most early-stage cases have favorable outcomes, melanomas can unexpectedly progress, highlighting the need for better risk prediction methods. This review examined the 31-gene expression profile (31-GEP) test, which analyzes the activity of 31 genes in melanoma tumors to predict the risk of metastasis. Data from multiple studies was systematically reviewed and analyzed to assess the 31-GEP test’s efficacy in predicting melanoma patient outcomes. Our findings suggest that the 31-GEP test may improve risk prediction when used alongside standard clinical and pathological assessments. By using 31-GEP, physicians may be better able to make more informed decisions about treatment and follow-up care, potentially improving outcomes for melanoma patients.

## 1. Introduction

Cutaneous melanoma (CM) represents a significant public health concern, ranking as the 5th most common cancer in the United States, with approximately 100,000 new cases diagnosed annually, and having an increasing worldwide incidence rate, with an estimated 325,000 new melanoma diagnoses being made in 2020 [1,2,3]. The incidence of cutaneous melanoma has steadily increased in populations of fair skin tone since the 1950s, with the primary drivers thought to be increased ultraviolet light exposure, whether through exposure to sunlight or indoor tanning, and an aging population [4,5,6]. While screening for melanoma and other skin cancers is not currently recommended by the United States Preventive Services Taskforce, an increase in public awareness and improved access to sunscreen may be related to an observed decline in the incidence of melanoma among certain populations, such as among younger Australians [7,8]. Despite advances in treatment modalities, CM continues to pose substantial morbidity and mortality risks, underscoring the critical importance of accurate risk stratification in guiding patient management and improving outcomes [9].

The current gold standard for melanoma staging and prognostication is the American Joint Committee on Cancer (AJCC) staging system, which primarily relies on clinicopathological features such as Breslow thickness, ulceration status, and lymph node involvement [10]. However, the limitations of this approach have become increasingly apparent, particularly in early-stage disease. This is evidenced by the increased mortality of patients with stage IIC melanoma compared to patients with IIIA. While it is well established that the risk of melanoma-associated mortality increases with greater depth of invasion of the primary lesion at the time of diagnosis, Baade et al. showed that in melanoma patients with decreased depth of invasion (<1.0 mm), case fatality was greater at follow-up between 5 and 20 years following diagnosis than within the first 5 years [11,12]. The fact that nearly 90% of melanomas are diagnosed in the early stages highlights the unpredictable nature of tumor behavior and the inadequacy of current staging systems in accurately stratifying and prognosticating melanoma patients according to disease risk [13].

This discrepancy between the initial staging and the ultimate patient outcome underscores the urgent need for more refined and accurate methods of risk stratification. The advent of molecular testing, particularly gene expression profiling (GEP), has emerged as a promising approach to address this clinical challenge. Although still in its early stages, GEP has the potential to provide a more comprehensive assessment of tumor biology, capturing the complex molecular landscape that drives melanoma progression and metastasis [14,15].

Currently, two GEP assays have gained traction for use in cutaneous melanoma: the 31-gene expression profile (31-GEP) test (DecisionDx-Melanoma, Castle Biosciences Inc.) in the United States and the MelaGenix test (NeraCare GmbH) in Europe, along with the Merlin test (SkylineDX) that incorporates the expression of eight genes along with clinicopathologic variables [15,16,17]. These tests aim to enhance prognostic accuracy beyond traditional clinicopathological evaluation, potentially enabling more personalized and effective management strategies. The 31-GEP test has been the subject of numerous studies evaluating its prognostic capabilities in melanoma. This test analyzes the expression of 31 genes involved in various aspects of melanoma biology, including cell differentiation, tumor microenvironment, and immune response [15,18,19]. Previous cohort studies have demonstrated the test’s ability to stratify patients into risk categories that correlate with outcomes such as recurrence-free survival, distant metastasis-free survival, and overall survival [15,20,21].

The integration of GEP testing into clinical practice represents a paradigm shift in melanoma management that carries the potential of advancing prognostication approaches beyond the traditional reliance on clinicopathological evaluation. This molecular approach offers insight into the complex biological heterogeneity of melanoma tumors, which may not be fully reflected in conventional staging systems. By providing a more nuanced understanding of tumor biology, GEP testing aims to address the limitations of current prognostic models, particularly in early-stage disease where the risk of progression can be highly variable and difficult to predict based on clinicopathological features alone.

Despite the growing body of literature supporting the prognostic accuracy of the 31-GEP in melanoma, questions remain regarding its clinical utility across diverse patient populations. To provide a comprehensive assessment of the current evidence of its prognostic performance, we conducted a systematic review and meta-analysis of studies evaluating the prognostic performance of the 31-GEP test for cutaneous melanoma.

By synthesizing the available evidence published in the past literature, the current meta-analysis aims to provide clinicians and researchers with a robust understanding of the current state of GEP testing’s clinical utility in melanoma prognostication. Our findings may inform clinical decision-making, guide the integration of molecular testing into melanoma management algorithms, and highlight areas for future research to further refine and validate gene expression profiling assays in melanoma care. As the field of precision oncology continues to evolve, the integration of molecular profiling techniques, like GEP, into clinical use has the potential to significantly improve risk stratification and, ultimately, patient outcomes in cutaneous melanoma prognostication and management. 

## 2. Genetics of Melanoma

The genetic landscape of melanoma is complex and diverse, with several key pathways being implicated in its pathogenesis [22,23]. The *MAPK* pathway plays a central role, with *BRAF* mutations occurring in approximately 50% of cutaneous melanomas, most commonly the *BRAF^V600E^* alteration. It should be noted, however, that many benign nevi harbor the same alteration without malignant transformation, reflecting the complex genetic characteristics of melanoma [23]. This discovery led to the development of *BRAF* inhibitors, such as vemurafenib and dabrafenib, which have significantly improved outcomes for patients with melanoma positive for *BRAF* mutations [24]. The *PI3K*-*AKT* pathway, often activated concurrently with *MAPK*, contributes to cell survival and metabolism, with *PTEN* loss being a common alteration that inhibits the pathway’s physiological functioning [25,26,27]. It should also be noted that recent studies have found that the use of *MEK* inhibitors in conjunction with *BRAF* inhibitors has significantly improved outcomes for patients with *BRAF*-positive cutaneous melanoma [28]. Cell cycle dysregulation, frequently due to *CDKN2A* inactivation, and telomere maintenance, through *TERT* promoter mutations, are also key events in melanoma development [29,30,31,32,33]. The complex interplay of these genetic alterations not only drives melanoma progression, but also influences treatment responses, underscoring the importance of comprehensive genomic profiling in guiding personalized therapeutic strategies [34,35].

## 3. Gene Expression Profiling

Gene expression profiling (GEP) has emerged as a valuable tool in oncology, offering insights into tumor biology, prognosis, and treatment response across various cancers [36,37,38,39]. In melanoma, the 31-GEP test (DecisionDx-Melanoma) has been developed to predict metastatic risk, aiming to provide a significant advancement in personalized melanoma management [15]. The 31-GEP test for cutaneous melanoma was developed to address the need for more accurate patient risk stratification. In developing the test, publicly available gene expression data from metastatic melanoma, along with genes from a validated GEP assay for uveal melanoma, were used to identify genes to be targeted for detection [40,41]. The 31-GEP assay consists of 31 genes in total, of which 28 are discriminant genes and three are control genes, with discriminant genes selected based on differential expression in metastatic tissue samples compared to non-metastatic samples [15]. By capturing the collective expression pattern of these genes, the 31-GEP assays aim to provide a more comprehensive assessment of tumor biology than traditional clinicopathological factors alone. The 31-GEP test classifies melanoma tumors into four risk classes: Class 1A (lowest risk), Class 1B and 2A (intermediate risk), and Class 2B (highest risk).

The development and validation of GEP tests, like the 31-GEP assay, represents a significant advancement in translational cancer research that bridges the gap between basic molecular biology and clinical application. These assays are designed to capture the collective impact of multiple genetic alterations and their downstream effects on cellular processes, providing a more holistic view of tumor behavior than single-gene assays. Ongoing research aims to determine how to best integrate 31-GEP testing with traditional clinicopathological evaluation. Recent studies have explored the application of machine learning algorithms to combine GEP risk classification with clinical variables, showing promise in improving risk stratification for sentinel lymph node biopsy [42,43,44]. These integrated approaches represent the next frontier in melanoma prognostication and treatment planning.

## 4. Methods

The present systematic review and meta-analysis were conducted in accordance with the Preferred Reporting Items for Systematic Reviews and Meta-Analyses (PRISMA) guidelines. [45]. The protocol was registered with the International Prospective Register of Systematic Reviews (PROSPERO, University of York, York, UK; protocol ID CRD42024581715). 

### 4.1. Information Source and Search Strategy

PubMed (U.S. National Library of Medicine, National Institutes of Health, https://pubmed.ncbi.nlm.nih.gov, accessed on 1 July 2024), Scopus (Elsevier https://www.scopus.com/search/form.uri, accessed on 1 July 2024), PsycINFO (American Psychological Association https://www.ebsco.com/products/research-databases/apa-psycinfo, accessed on 1 July 2024), and Cochrane (Cochrane Library, Cochrane, https://www.cochranelibrary.com/search, accessed on 1 July 2024) databases were systematically searched from inception through 1 July 2024 to identify English-language articles examining the performance of the GEP test among melanoma patients. Keywords used in the search strategies include “Cutaneous Melanoma”, “Primary Melanoma”, and “Gene expression”. Each database’s respective final search strategy is outlined in Supplementary Table S1.

### 4.2. Screening and Study Selection

**Figure 1:**
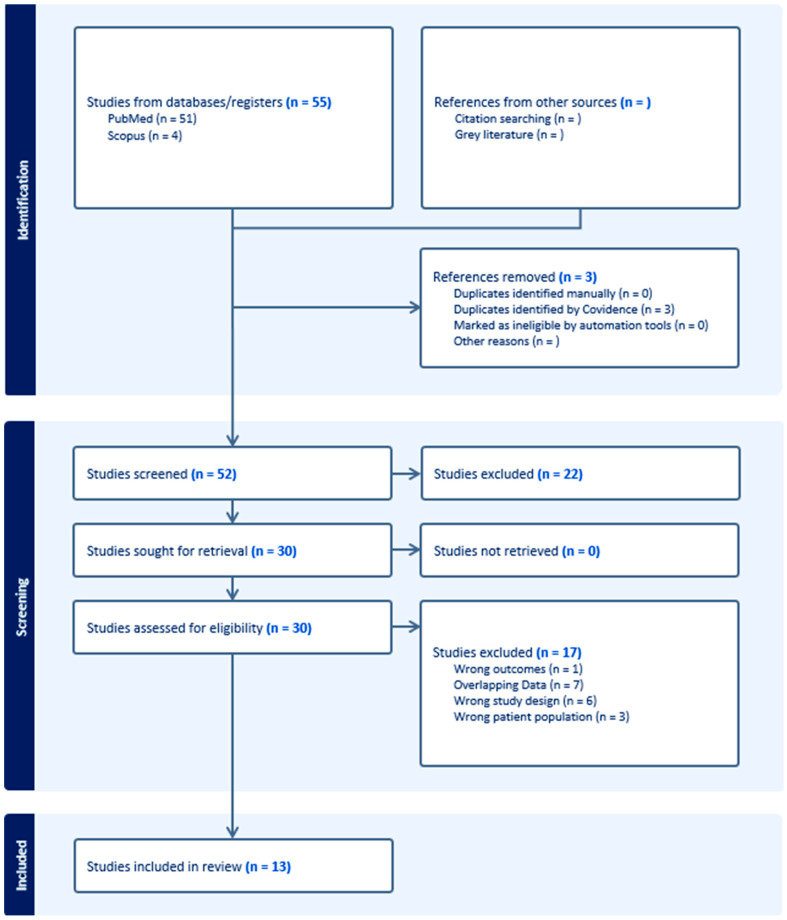
PRISMA Diagram showing included studies.

Covidence (Veritas Health Innovation, Melbourne, Australia), software that facilitates reviewer collaboration in screening articles was used to review the studies captured by the search strategies. The following inclusion criteria were applied to reviewed articles: (1) publications were peer-reviewed, (2) the article was available in English, and (3) articles included data on GEP class and at least one survival-based metric with follow-up data obtained over a minimum of 3 years. Exclusion criteria used while reviewing articles included: (1) not published in a peer-reviewed journal, (2) no English translation available, (3) no full-text manuscript available, (4) SR-MA methodology, (5) not reporting any survival-related outcome with a follow-up time of at least 3 years, and (6) not differentiating results by GEP class. Two reviewers, R.A.D. and S.I.N, independently screened titles and abstracts for relevance. Conflicts resulted in a conversation to achieve consensus and in cases of continued conflict, discussion with a third reviewer, S.A.N. Thereafter, R.A.D. and S.I.N. reviewed the full texts of articles that were found to be eligible, with S.A.N. resolving any conflicts on studies to be included in the final meta-analysis. The PRISMA diagram is displayed in Figure 1. 

### 4.3. Data Extraction

Two reviewers, R.A.D. and S.I.N., independently extracted data from included studies to ensure accuracy. Data extracted included the author, year of publication, years examined by the study, data source used, mean age, gender distribution, size of study population (total and by GEP classification), reported 3 and/or 5-year survival-related metrics (Disease-free survival, recurrence-free survival, distant metastasis-free survival, overall survival), and sentinel lymph node biopsy results, demographic variables, and tumor covariates. Tumor covariates included Breslow thickness, presence of ulceration, and location. Any conflicts were resolved by discussion with S.A.N.

### 4.4. Quality Assessment

**Figure 2:**
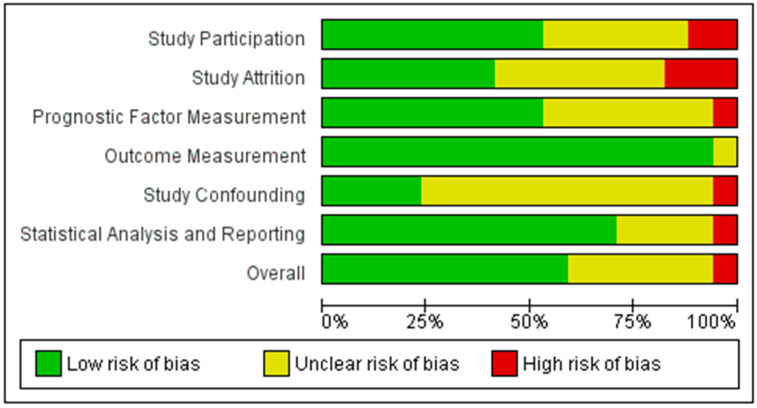
Overall risk of bias assessment.

Level of evidence and risk of bias of the included studies were assessed per the Oxford Center for Evidence-Based Medicine guidelines and the Quality in Prognosis studies (QUIPS) tool, respectively [46,47]. The risk of bias assessment was performed independently by two authors, R.A.D. and S.I.N. Any disagreements were resolved via discussion with a third author, S.A.N. The QUIPS tool assesses bias in six domains: study participation, study attrition, prognostic factor measurement, outcome measurement, study confounding, and statistical analysis/reporting. Each domain is reported as being at a low, unclear, or high risk of bias, as shown in Figure 2.

### 4.5. Statistical Analysis

Meta-analysis of proportions (sex, tumor characteristics, survival outcomes) and meta-analysis of single means (age, Breslow thickness) was performed using R version 4.4.0 (R Foundation for Statistical Computing, Vienna, Austria). Each measure (mean/proportion (%) and 95% confidence interval [CI]) was weighted according to the number of patients affected. As some studies reported the outcomes using the median (first quartile, third quartile), the quantile estimation (QE) method was deployed to calculate the pooled estimates [48]. Due to high levels of heterogeneity and the small number of studies consistently reporting survival outcomes for a particular GEP class at identical time points (3- or 5-year), random effects estimates were used throughout. Publication bias was evaluated by visual inspection of the funnel plot and Begg’s rank correlation test. Finally, potential publication bias was evaluated by visual inspection of the funnel plot and both Begg’s test and Egger’s regression test, which statistically examines the asymmetry of the funnel plot. Begg’s test assesses the significance of the correlation between the ranks of the standardized effect sizes and the ranks of their variances [49]. Egger’s test is a test for the Y intercept = 0 from a linear regression of normalized effect estimate (estimate divided by its standard error) against precision (reciprocal of the standard error of the estimate) [50]. A *p*-value of <0.05 was considered to indicate a significant difference for all statistical tests. 

## 5. Results

### 5.1. Included Studies 

Of the 52 unique articles identified, 13 were determined to meet the inclusion criteria for the present review. The study selection process is outlined in Figure 1. A summary of the included studies is presented in Supplementary Table S1. The reported data were derived from GEP testing done between 1998 and 2020. Most studies used either prospective institutional data (6/13) or retrospective data (5/13), with two studies using the SEER 2009–2018 databases. While Podlipnik et al.’s (2024) SEER database portion of the study was used, it was determined that their institutional data had been previously reported and was thus excluded from the meta-analysis [51].

**Figure 3:**
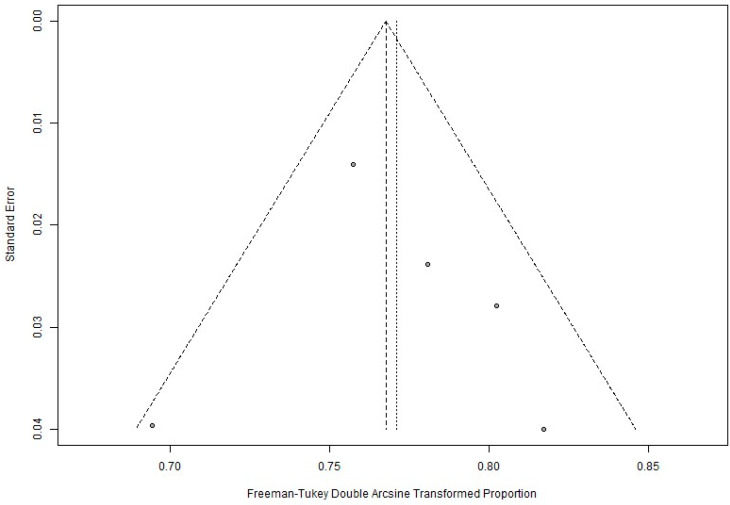
Illustrative funnel plot assessing for publication bias in location (extremity) among all included patients.

Critical appraisal of these prognostic studies (Figure 2) indicated an overall acceptably low risk of bias with the potential sources of bias being most pronounced due to study confounding, attrition, and participation. As shown in the funnel plot (Figure 3) with Begg’s test, all studies were inside the funnel with little asymmetry and Egger’s test was non-significant, suggesting a low risk of publication bias.

### 5.2. Melanoma Patient Cohort Characteristics

As summarized in Table 1, in the overall population, 47.6% (95% CI: 37.2–58.1%) of tumors were ulcerated, per the findings of 12 studies including 10,073 patients. The mean Breslow thickness was 3.94 mm (95% CI: 2.6–5.2 mm), derived from 13 studies with 14,760 patients. Regarding tumor location, 77.1% (95% CI: 72.2–82.0%) were found on the extremities, 63.8% (95% CI: 16.5–100%) on the head and neck, and 60.2% (95% CI: 52.5–68%) on the trunk. Tumor staging data showed that 53.2% (95% CI: 27.8–78.6%) were T1, 50.5% (95% CI: 15.9–85%) were T2, and 35.0% (95% CI: 10.7–59.3%) were T3.

In Class 1 tumors, 41.5% (95% CI: 20–63.1%) were ulcerated, while a higher proportion of Class 2 tumors (67.6%, 95% CI: 45.6–89.6%) showed ulceration. Class 1A tumors had the lowest ulceration rate at 14.1% (95% CI: 4.5–23.6%), with a mean Breslow thickness of 1.56 mm (95% CI: 0.84–2.3 mm). In contrast, Class 2B tumors had the highest mean Breslow thickness at 3.74 (95% CI: 1.64–5.84).

### 5.3. Survival Outcomes

As shown in Table 2, the survival outcomes varied significantly between GEP classes across different metrics. The 5-year melanoma-specific survival (MSS) rate for the overall population was 97.5% (95% CI: 86.5–99.6%). When stratified by GEP class, Class 1A showed the highest 5-year MSS at 99.8% (95% CI: 98–100%), followed by Class 1B/2A at 97.6% (95% CI: 92.4–99.3%), and Class 2B at 83.4% (95% CI: 66.5–92.7%). Three-year overall survival rates also demonstrated clear differences between classes, with Class 1 patients showing a rate of 96.1% (95% CI: 95.5–96.6%) compared to 82.2% (95% CI: 81.1–83.2%) for Class 2 patients. Similar patterns were observed in recurrence-free survival (RFS) and distant metastasis-free survival (DMFS) rates. The 5-year RFS rate for Class 1A was 95.0% (95% CI: 91.8–97.0%), significantly higher than the 50.5% (95% CI: 42.4–58.7%) RFS rate observed for Class 2B. DMFS rates at 5 years showed a similar trend, with Class 1A at 98.0% (95% CI: 96.1–98.9%) compared to 62.4% (95% CI: 52.5–71.4%) for Class 2B. These results consistently demonstrate better outcomes for Class 1 and 1A compared to Class 2 and 2B across various survival metrics.

## 6. Discussion

Our meta-analysis, encompassing 13 studies with a total of 14,760 patients, provides comprehensive evidence for the prognostic value of the gene expression profile (GEP) assay in the risk stratification of cutaneous melanoma [15,20,51,52,53,54,55,56,57,58,59,60,61]. We found significant differences in survival outcomes between GEP classes, with Class 1A consistently showing better outcomes compared to Class 2B across various survival metrics. Our findings are largely consistent with and expand upon the results of the previous systematic review and meta-analysis authored by Greenhaw et al. (2020), which included 1479 patients from three studies in addition to a novel cohort [62].

The strong prognostic value of the 31-GEP assay demonstrated in this meta-analysis suggests a potential role in personalized management strategies for patients with cutaneous melanoma. For early-stage melanoma, a Class 1A result might support less intensive follow-up, while a Class 2B result might prompt more frequent follow-up or consideration of adjuvant therapy. However, it is crucial to emphasize that the GEP test should be used in conjunction with established clinicopathologic factors and that currently the National Comprehensive Cancer Network guidelines for cutaneous melanoma note that the test was developed and initially validated using a relatively high-risk group of patients [12]. Additionally, a recent consensus document produced by the Melanoma Research Foundation found that panelists consistently supported a routine approach based on histopathological features rather than GEP results based on available evidence [63]. Further, the NCCN guidelines identify that when compared to all available GEP tests for melanoma, there is a relatively small amount of overlap in genes, perhaps reflecting a still incomplete understanding of the complex interplay of genes ultimately resulting in metastasis [15,64,65,66].

The integration of GEP testing into clinical practice requires careful consideration of its strengths and limitations. While the test offers valuable prognostic information, it should be viewed as a complementary tool rather than a replacement for established standard clinicopathological assessment. The optimal use of GEP testing likely involves a comprehensive approach that combines molecular data with an assessment of patient risk factors and patient preferences, in addition to the clinician’s best judgment [67,68,69].

Furthermore, the potential impact of GEP testing on clinical decision making extends beyond individual patient management. At a broader level, the ability to more accurately stratify patients based on their risk of recurrence and metastasis could inform the design and conduct of clinical trials. For instance, enriching high-risk populations in adjuvant therapy trials could lead to more efficient study designs and potentially accelerate the development of new treatments for melanoma.

This meta-analysis has several strengths, including a comprehensive literature search, a large overall sample size, and the inclusion of studies from diverse geographic regions. We performed a rigorous quality assessment and explored potential sources of heterogeneity through subgroup analyses. However, several important limitations should be considered when interpreting the results.

### Limitations

A primary limitation is the small number of studies available for each GEP class per survival-related outcome, with only 2–4 studies being used for most subgroup analyses. This limited number of studies restricts the precision and reliability of these estimates, and we observed substantial heterogeneity among studies for all outcomes, with I² values as high as 99.4% for 5-year melanoma-specific survival. While we used random-effects models to account for this, the high degree of heterogeneity introduced uncertainty into our analyses and may limit the generalizability of our findings.

Furthermore, very few studies mentioned the specific treatments used for patients beyond primary surgical management. This lack of information on treatment modalities employed is particularly significant considering that checkpoint inhibitors and other immunotherapies were introduced for adjuvant melanoma treatment over the period that these samples were collected and GEP assays were tested [70]. The evolution of melanoma treatment during this time could have influenced outcomes independently of GEP classification, potentially confounding our results. Additionally, it should be noted that, particularly for T1 patients, a 5-year follow-up time may not adequately capture the entire risk of recurrence [11,12]. 

Another limitation stems from the GEP test’s categorical risk stratification, which conflicts with the Melanoma Prevention Working Group’s recommendations for GEP tests to be reported as continuous variables [70]. This categorical approach may lead to loss of information and reduced precision in risk stratification. Our analysis, as well as the analyses of many of the included studies primarily focused on the comparison between Class 2B and Class 1A results, representing the extremes of the risk spectrum. While this provides valuable information about the test’s ability to discriminate between high and low-risk patients, it does not fully capture the nuances of risk stratification for patients with intermediate results (i.e., Class 1B or 2A).

Research is ongoing to address some of these limitations. Recently, machine learning algorithms incorporating both the GEP class along with clinicopathologic variables have been developed that analyze the performance of a machine learning model utilizing clinicopathologic characteristics as well as GEP assay results [42,43,44,71,72]. Several of these have demonstrated that a low-risk result (<5% predicted probability of sentinel lymph node metastasis) has a very high negative predictive value for sentinel lymph node biopsy. However, these approaches are still in the early stages of validation and were not included in our meta-analysis.

Despite these limitations, our study provides valuable insights into the prognostic value of the 31-GEP assay in the risk stratification of cutaneous melanoma. Future prospective studies with larger sample sizes, more comprehensive treatment information, and longer follow-up periods will be crucial to further validate these findings and clarify the optimal clinical use of the 31-GEP assay.

## 7. Conclusions

This meta-analysis provides further supporting evidence for the prognostic value of the 31-gene expression profile test for cutaneous melanoma. Patients with a Class 2B result have consistently worse outcomes across multiple survival measures compared to those with a Class 1A result. These findings support the prognostic capability of the 31-GEP test in patients with cutaneous melanoma. However further evidence, ideally by way of randomized-control trials evaluating long-term outcomes of patients with GEP-informed testing is necessary to further define the possible role of the 31-GEP test in the management of patients with melanoma.

The consistency of the test’s prognostic value across different subgroups suggests broad applicability in melanoma management. However, the observed heterogeneity between studies underscores the need for cautious interpretation and further validation, particularly through large-scale prospective studies. This is especially relevant considering that for many early-stage melanoma patients, nearly 20% of recurrences may occur after five years or more [73]. Additionally, further research on the costs and logistics of incorporating GEP into the clinical management of melanoma is necessary [74,75].

As our understanding of melanoma biology continues to evolve and new treatment options emerge, molecular prognostic tools like the 31-GEP assay are likely to become increasingly clinically important. The ability to more accurately stratify patients based on their risk of recurrence and metastasis has the potential to significantly improve personalized care strategies, optimizing the balance between treatment efficacy and quality of life for patients with cutaneous melanoma. In conclusion, while further research is needed to fully define its optimal clinical use, in terms of clarifying which patients may benefit most from the prognostic insight provided by the 31-GEP assay as well as how best to integrate these findings into treatment and management algorithms, the 31-GEP assay represents a promising tool for enhancing risk stratification and potentially improving patient outcomes in the management of this challenging malignancy.

## Figures and Tables

**Table 1:** Summary of tumor characteristics in included studies.

Variable	Subclass	N Studies	Total Patients	Random Effects Estimate (95% CI)
Ulcerated	Total	12	10,073	47.6% (37.2–58.1%)
T1	Total	3	6193	53.2% (27.8–78.6%)
T2	Total	4	10,880	50.5% (15.9–85%)
T3	Total	3	5229	35.0% (10.7–59.3%)
Mean Breslow (mm)	Total	13	14,760	3.94 (2.6–5.2)
Extremity	Total	5	2335	77.1% (72.2–82.0%)
Head and Neck	Total	6	3320	63.8% (16.5–100%)
Trunk	Total	5	2335	60.2% (52.5–68%)
Ulcerated	Class 1	4	686	41.5% (20–63.1%)
Extremity	Class 1	3	618	73.0% (59.7–86.3%)
Head and Neck	Class 1	3	618	45.7% (34.9–56.5%)
Trunk	Class 1	3	618	65.0% (47.2–82.7%)
Ulcerated	Class 2	5	404	67.6% (45.6–89.6%)
T1	Class 2	3	287	37.9% (0–81.7%)
T2	Class 2	3	287	61.6% (40.4–82.8%)
T3	Class 2	3	287	53.1% (41.6–64.5%)
T4	Class 2	3	287	50.1% (17.6–82.5%)
Extremity	Class 2	3	242	76.3% (55.9–96.6%)
Head and Neck	Class 2	3	242	49.4% (30.7–68.0%)
Trunk	Class 2	3	242	57.4% (44.3–70.6%)
Ulcerated	Class 1A	4	5754	14.1% (4.5–23.6%)
Mean Breslow (mm)	Class 1A	5	5926	1.56 (0.84–2.3)
Extremity	Class 1A	3	1385	74.7% (64.3–85.2%)
Head and Neck	Class 1A	3	1385	45.8% (41–50.6%)
Trunk	Class 1A	3	1385	62.3% (55.9–68.7%)
Ulcerated	Class 2B	4	449	70.1% (11.6–100%)
Mean Breslow	Class 2B	5	1496	3.74 (1.64–5.84)
T1	Class 2B	3	407	37.6% (14–61.1%)
T2	Class 2B	3	407	53.3% (15–91.7%)
Extremity	Class 2B	3	239	74.6% (70.3–78.9%)
Head and Neck	Class 2B	3	239	51.9% (35.8–68.1%)
Trunk	Class 2B	3	239	57.7% (42.2–73.1%)

**Table 2:** Summary of Random Effects Estimates for Survival-Related Outcomes.

Outcome	Number of Studies	Total Patients	Random Effects Estimate (95% CI)	I2
Total 5-year Melanoma Specific Survival %	2	6552	97.5% (86.5–99.6%)	99.4%
Class IA 5-year Melanoma Specific Survival %	4	7513	99.8% (98.0–100%)	63.7%
Class IB/IIA 5-year Melanoma Specific Survival %	3	6990	97.6% (92.4–99.3%)	98.6%
Class IIB 5-year Melanoma Specific Survival %	4	7513	83.4% (66.5–92.7%)	99.5%
Class I 3-year Overall Survival %	2	5010	96.1% (95.5–96.6%)	13.1%
Class II 3-year Overall Survival %	2	5010	82.2% (81.1–83.2%)	0.0%
Class I 3-year Recurrence-free survival %	2	482	95.4% (93.2–97.0%)	6.9%
Class II 3-year Recurrence-free survival %	2	482	57.2% (43.8–69.6%)	93.5%
Class IA 3-year Recurrence-free survival %	2	1283	98.5% (97.7–99.1%)	0.0%
Class IA 5-year Recurrence-free survival %	3	2085	95.0% (91.8–97.0%)	91.6%
Class IB 3-year Recurrence-free survival %	2	1283	94.0% (92.6–95.2%)	0.0%
Class IB 5-year Recurrence-free survival %	2	1647	83.3% (74.4–89.5%)	96.8%
Class IIA 3-year Recurrence-free survival %	2	1283	81.1% (60.4–92.3%)	98.3%
Class IIA 5-year Recurrence-free survival %	2	1647	82.3% (65.6–91.9%)	98.9%
Class 2B 3-year Recurrence-free survival %	2	1283	48.1% (37.6–58.8%)	92.3%
Class IIB 5-year Recurrence-free survival %	3	2085	50.5% (42.4–58.7%)	93.4%
Class I 3-year Distant Met-free survival %	2	482	98.0% (94.4–99.3%)	57.7%
Class I 5-year Distant Met-free survival %	2	740	88.7% (79.0–94.2%)	94.6%
Class II 3-year Distant Met-free survival %	2	482	72.5% (61.2–81.5%)	91.6%
Class II 5-year Distant Met-free survival %	2	740	53.6% (43.8–63.1%)	91.7%
Class IA 3-year Distant Met-free survival %	2	1283	99.3% (98.7–99.6%)	0.0%
Class IA 5-year Distant Met-free survival %	3	2085	98.0% (96.1–98.9%)	81.5%
Class IB 3-year Distant Met-free survival %	2	1283	99.2% (85.7–100%)	0.0%
Class IB 5-year Distant Met-free survival %	2	1647	87.1% (79.8–92.1%)	96.1%
Class IIA 3-year Distant Met-free survival %	2	1283	86.3% (69.6–94.6%)	97.8%
Class IIA 5-year Distant Met-free survival %	2	1647	86.8% (72.2–94.4%)	98.7%
Class 2B 3-year Distant Met-free survival %	2	1283	66.6% (63.9–69.1%)	73.4%
Class IIB 5-year Distant Met-free survival %	3	2085	62.4% (52.5–71.4%)	95.5%

## Data Availability

The extracted data supporting the conclusions of this article will be made available by the authors on request.

## Supplementary Materials

The following supporting information can be downloaded at: https://www.mdpi.com/article/10.3390/cancers16213714/s1, Search Strategy; Table S1: Summary of Included Studies; Table S2: Summary of all genes in 3 gene expression profiling tests available for use in cutaneous melanoma.
