# Supplementary material for: The Prognostic Value of the 31-Gene Expression Profile Test in Cutaneous Melanoma: A Systematic Review and Meta-Analysis
Source: Cancers (Basel). 2024 Nov 4;16(21):3714. doi: 10.3390/cancers16213714 (PMC11545106; doi:10.3390/cancers16213714)
Supplement: Supplementary file 1 [file cancers-16-03714-s001.zip › cancers-3271668-supplementary.pdf]

Search Strategy:

- Pubmed
  - ("cutaneous melanoma" OR "primary melanoma") AND ("expression profil*" OR "gene expression" OR "gene-signature" OR "31-gene" OR "31-GEP") AND "Castle"
    - 7/1/2024: 51 Results
- CINAHL
  - ("cutaneous melanoma" OR "primary melanoma") AND ("expression profile" OR "gene expression" OR "gene-signature" OR "31-gene" OR "31-GEP") AND "Castle"
    - 7/1/2024: 0 results
- SCOPUS
  - ("cutaneous melanoma" OR "primary melanoma") AND ("expression profile" OR "gene expression" OR "gene-signature" OR "31-gene" OR "31-GEP") AND "Castle"
    - 7/1/2024: 4 Results
- Cochrane
  - ("cutaneous melanoma" OR "primary melanoma") AND ("expression profil*" OR "gene expression" OR "gene-signature" OR "31-gene" OR "31-GEP") AND "Castle"
  - 7/1/2024: 0 results

Table S1: Summary of Included Studies

Author (Year)	Years	Data Source	Design	OCEBM	GEP N	GEP 1 N	GEP 2 N	GEP 1A N	GEP 1B N	GEP 1B/2AN	GEP 2A N	GEP 2B N	Factors examined; note that all studies included tumor characteristics
Arnot 2021	2014-2020	OHSU	Prospective Cohort	4	383	251	132	172	79	136	57	75	- SLNB, sex, age
Bailey 2023	2009-2018	SEER	Big Database	4	4687	3641	1046	3174	467	845	378	668	- 3-year Overall survival, melanoma specific survival
Berger 2016	2013-2015	6 institutions	Retrospective	4	156	95	61						- Stage, SLNB
Gerami 2015	1998-2009	7 institutions	Retrospective, archived samples	4	217	76	141						- SLNB, 5-year overall survival
Hsueh 2021	2013-2019	11 institutions	Prospective study	4	323	252	71						- Sex, age, stage, 3-year overall survival, recurrence-free survival, distant metastasis-free survival

Hyams 2021	2015-2017	1 surgical center	Prospective study	4	112	66	46						- Sex, age, stage, SLNB
Jarell 2021	2014-2019	1 dermatology, 2 surgery centers	Retrospective chart review	4	438			248		94		96	- Sex, age, stage, SLNB, 5-year MSS, RFS, DMFS
Keller 2019	2013-2015	Patients	Prospective	4	159	117	42						- Sex, age, 3-year RFS, DMFS,
Podlipnik 2022	2015-2016	5 melanoma referral centers	Prospective study	4	86	53	33	40	13	25	12	21	- Sex, age, stage
Podlipnik 2024 A	2013-2018	SEER 2013-2018	Retrospective	4	5651			4526		865		260	- Sex, age, 5-year MSS
Thorpe 2022	Unspecified	9 Mohs micrographic surgery	Prospective study	4	1124	1,011	113	940	71	112	41	72	- Sex, age, SLNB, 3+5 year RFS, DMFS
Wisco 2022	1998-2016	Multiple centers, unspecified number	Retrospective	4	901			402		195		304	- Age, stage, 5-year MSS,
Zager 2018	2000-2014	16 institutions	Retrospective	4	523	314	209						- Age, stage, SLNB, 5-year MSS, RFS, DMFS

Table S2: Summary of all genes in 3 gene expression profiling tests available for use in cutaneous melanoma.

31-GEP	**Melagenix (11-GEP)**	**Skygenix**
BRCA1-associated protein-1; *BAP1*	Keratin 9; *KRT9*	Melanoma antigen recognized by T cells 1*MLANA*
Matrix Gla protein; *MGP*	Dermcidin; *DCD*	Growth differentiation factor 15; *GDF15*
Secreted phosphoprotein 1; *SPP1*	Prolactin-inducible protein; *PIP*	Interleukin 8; *CXCL8*
Chemokine (C-X-C motif) ligand 14; *CXCL14*	Secretoglobin family 1D member 2; *SCGB1D2*	Lysyl oxidase homolog 4; *LOXL4*
Chloride channel accessory 2; *CLCA2*	Secretoglobin family 2A member 2; *SCGB2A2*	TGF-β receptor type 1; *TGFBR1*
S100 calcium-binding protein A8; *S100A8*	Collagen type VI alpha 6 chain; *COL6A6*	Integrin-β3; *ITGB3*
B-cell translocation gene 1, antiproliferative; *BTG1*	Guanylate Binding Protein 4*;* *GBP4*	Tissue-type plasminogen activator; *PLAT*
Sin3A-associated protein, 130 kDa; *SAP130*	Kelch Like Family Member 41; *KLHL41*	Glia-derived nexin; *SERPINE2*
Arginase 1; *ARG1*	Esophageal Cancer-Related Gene 2; *ECRG2*	
Keratin 6B; *KRT6B*	Hes Family BHLH Transcription Factor 6; *HES6*	
Gap junction protein, alpha 1, 43 kDa; *GJA1*	Mucin-7; *MUC7*	
Inhibitor of DNA binding 2, dominant negative helix-loop-helix protein; *ID2*		
Eukaryotic translation initiation factor 1B; *EIF1B*		
S100 calcium-binding protein A9; *S100A9*		
Cellular retinoic acid binding protein 2; *CRABP2*		
Keratin 14; *KRT14*		
Roundabout, axon guidance receptor, homolog 1 (Drosophila); *ROBO1*		
RNA-binding motif protein 23; *RBM23*		

31-GEP	**Melagenix (11-GEP)**	**Skygenix**
Tumor-associated calcium signal transducer 2;*TACSTD2*		
Desmocollin; *DSC1*		
Small proline-rich protein 1B; *SPRR1B*		
Tripartite motif containing 29; *TRIM29*		
Aquaporin 3 (Gill blood group); *AQP3*		
Tyrosinase-related protein 1; *TYRP1*		
Periplakin; *PPL*		
Leukotriene A4 hydrolase; *LTA4H*		
Cystatin E/M; *CST6*		
